# Analysis of Insect Pests in an 18th-Century Historical Pharmacy: A Case Study in Ferrara

**DOI:** 10.3390/insects12090839

**Published:** 2021-09-18

**Authors:** Loren Anna Palazzo, Chiara Beatrice Vicentini, Maria Gabriella Marchetti, Marilena Leis, Milvia Chicca, Chiara Scapoli, Teresa Bonacci, Marco Pezzi

**Affiliations:** 1 Department of Life Sciences and Biotechnology, University of Ferrara, Via L. Borsari 46, 44121 Ferrara, Italy; lorenanna.palazzo@edu.unife.it (L.A.P.); chiara.vicentini@unife.it (C.B.V.); gabriella.marchetti@unife.it (M.G.M.); marilena.leis@unife.it (M.L.); milvia.chicca@unife.it (M.C.); chiara.scapoli@unife.it (C.S.); 2 Department of Biology, Ecology and Earth Sciences, University of Calabria, Via P. Bucci, Arcavacata di Rende, 87036 Cosenza, Italy; teresa.bonacci@unical.it

**Keywords:** biodeterioration, drugstore beetle, equipment, furniture, historical pharmacy, powderpost beetle, varied carpet beetle

## Abstract

**Simple Summary:**

Historical pharmacies are cultural and scientific assets, and 482 of them have been catalogued thus far in Italy. The Navarra-Bragliani Pharmacy in Ferrara has valuable wood furniture dating back to the 18th century. In order to monitor and preserve it, a study on biodeteriogenic insects was carried out for the first time on the furnishing of this pharmacy, collecting insect samples and examining them by stereomicroscopy and scanning electron microscopy. The presence of three species of Coleoptera was detected—the first one specialised to attack wood materials, the second one polyphagous, and the third one feeding on materials of animal origin. In addition to their use in the preservation of the precious furnishing of the Navarra-Bragliani Pharmacy, the results of this study could be useful for similar studies on other ancient pharmacies, in order to protect these valuable cultural and scientific assets.

**Abstract:**

Historical pharmacies are valuable artistic, cultural, and scientific assets. In Italy, 482 historical pharmacies have been catalogued thus far, among which is the Navarra-Bragliani Pharmacy in Ferrara, whose wood furniture dates back to the 18th century. A study on insects causing biodeterioration was carried out for the first time on this valuable asset in order to monitor and preserve it. Insect samples were collected during surveys and examined by stereomicroscopy and scanning electron microscopy, using taxonomical keys for identification. The results revealed the presence of three species of Coleoptera—the first one specialised to attack wood materials, the second one polyphagous, and the third one feeding on materials of animal origin. The data obtained in this investigation may be useful for the preservation of the precious furnishing of the historical Navarra-Bragliani Pharmacy for performing similar studies on other ancient pharmacies aimed to protect these valuable cultural and scientific assets.

## 1. Introduction

Historical pharmacies are pharmaceutical establishments characterised by the historical and artistic interest that qualify them as cultural assets worthy of conservation. Given their artistic and cultural relevance, historical pharmacies are under the protection of the Soprintendenze (Superintendencies) of the Italian Ministry of Culture. According to data provided by Accademia Italiana di Storia della Farmacia (AISF) (Italian Academy of History of Pharmacy), 482 historical pharmacies have been catalogued until July 2021 in the Italian territory, and of these, 260 have been described in detail in the ‘Calendari’. The ‘Calendari’ are annual Special Issues published by AISF, cataloguing and describing each year by text and images 13 Italian historical pharmacies [1]. Among historical pharmacies known to date back to the 18th century is the Farmacia Navarra-Bragliani (Navarra-Bragliani Pharmacy) in Ferrara. Historical pharmacies are relevant cultural assets because they contain ancient equipment, books, jars, and furniture witnessing the history of apothecary and pharmaceutical arts. However, most of the ancient equipment and furniture (especially wood, parchment, paper, and jar contents) are subject to deterioration, therefore at risk of losing forever its historical and artistic importance. Among the aims of AISF is the protection of the historical and artistic heritage of ancient Italian pharmacies and their popularisation in the media.

**Figure 1:**
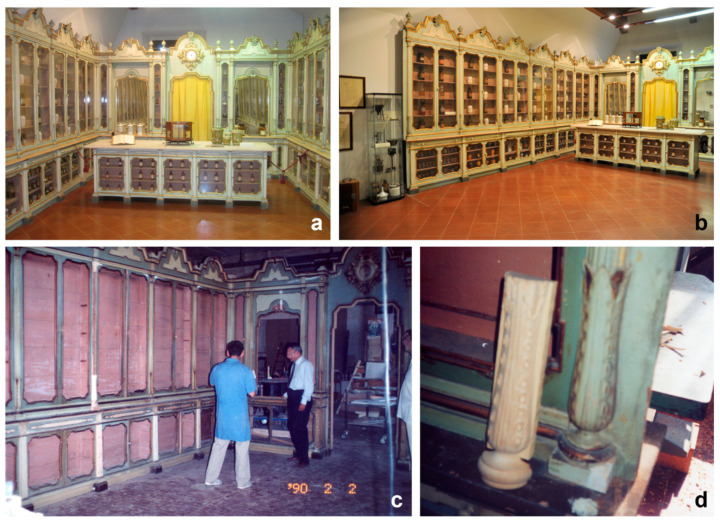
Historical Navarra-Bragliani Pharmacy in Ferrara (Italy): (**a**) overview of the elegant 18th-century furnishing composed of six wood cabinets on each side and a counter with four cabinets and two mirrors on the centre; (**b**) overview of the north side of the pharmacy; (**c**) restoration of the damaged furnishing in the 1990s (courtesy of Conservation Laboratory Maurizi, Montecosaro, Macerata, Italy); (**d**) detail of a reconstructed cabinet decoration (courtesy of Conservation Laboratory Maurizi, Montecosaro, Macerata, Italy).

Biological agents that may cause serious deterioration to cultural heritage include insects and fungi. A study of insect biodeterioration on furniture and equipment of the Navarra-Bragliani Pharmacy (Figure 1a,b), now belonging to Sistema Museale di Ateneo (SMA) (University Museum System) (Ferrara, Italy), was undertaken at the Department of Life Sciences and Biotechnology of the University of Ferrara.

## 2. Study Site

The valuable furnishing of the Navarra-Bragliani Pharmacy, which was established in 1738, dates back to the 18th century (Figure 1a,b). The first owner of the pharmacy was Giovanni Battista Nannini, followed by Carlo Bosi and Filippo Navarra. The pharmacy, together with its furnishing, was originally located in the central part of Ferrara, facing the world-famous Este Castle, and was a popular meeting place for social, political, and sports discussions. The place was so renowned that one of the short stories included in ‘Cinque storie ferraresi’ (Five stories of Ferrara), published in 1956 by the novelist and poet Giorgio Bassani, was set in the Navarra-Bragliani Pharmacy. The short story by Bassani, ‘Una notte del ‘43’ (A night in 1943), was also the subject of a film (‘La lunga notte del ‘43’, The long night of ‘43) directed in 1960 by Florestano Vancini.

The last owner of the pharmacy, Alessandro Bragliani, donated the historical furniture in 1977 to the University of Ferrara. The furniture was temporarily stored in the cloister of the monastery ‘Santa Maria delle Grazie’, belonging to the University. Unfortunately, the cloister, in itself under restoration, was not a suitable place, and the furniture suffered severe damages over the years, due to humidity and seasonal temperature changes.

In the 1990s, the damaged furniture was finally restored with the collaboration of local Lions Clubs, based also on the frames of the 1960 film. During the restoration, the elegant original colours of the wooden cabinets were discovered (Figure 1c,d). They had been originally painted in grey-blue colour, with ivory in the protruding parts, mostly intact. The gilded friezes decorating the tops of the display cabinets were also restored, together with the mirrors, a scenic backdrop to the refined shelving (Figure 1a,b). The restored furniture was finally located in the refectory of the cloister and opened to the public in 2011, within the SMA, enriched with historical instruments previously belonging to other ancient pharmacies [2,3,4,5,6].

The historical furniture of the Navarra-Bragliani Pharmacy, distributed along three sides of the cloister room, includes six lateral cabinets on each side (north and south) and four small cabinets and two mirrors on the east side behind the counter, which originally granted access to the backroom, the galenic formulation laboratory (Figure 1a,b).

## 3. Materials and Methods

The study on biodeterioration began with two surveys carried out on 14 February and 13 March 2019, during which photographic material was collected using a Canon digital camera (PowerShot A40, Canon, Milan, Italy). When damage or traces of biodeteriogenic insects were detected, samples were collected using laboratory and entomological tweezers or brushes and dissection needles. The collected samples were placed in sterile plastic containers with caps of various sizes or in clean Petri dishes. Each container was carefully labelled in order to precisely identify the sampling location. The samples were transferred to the Department of Life Science and Biotechnology and sorted under a stereomicroscope, to eliminate residues unrelated to biodeteriogenic activity. The samples relevant for the investigation were numbered and photographed. Some of them were identified by stereomicroscope observations, and those that could not be identified by stereomicroscopy were prepared for scanning electron microscopy (SEM) and identified using taxonomical keys and specialised books [7,8,9,10,11,12].

Since the samples were already dehydrated, they were directly fixed for SEM on aluminium stubs using a double-sided adhesive containing graphite. Subsequently, the samples were coated with a layer of gold and palladium in an Edwards S150 Sputter Coater metalliser (HHV Ltd., Crawley, UK). Observations were conducted at the Electron Microscopy Centre of the University of Ferrara using a Zeiss EVO 40 SEM (Milan, Italy). Images by SEM were acquired and used to identify the samples.

## 4. Results and Discussion

**Figure 2:**
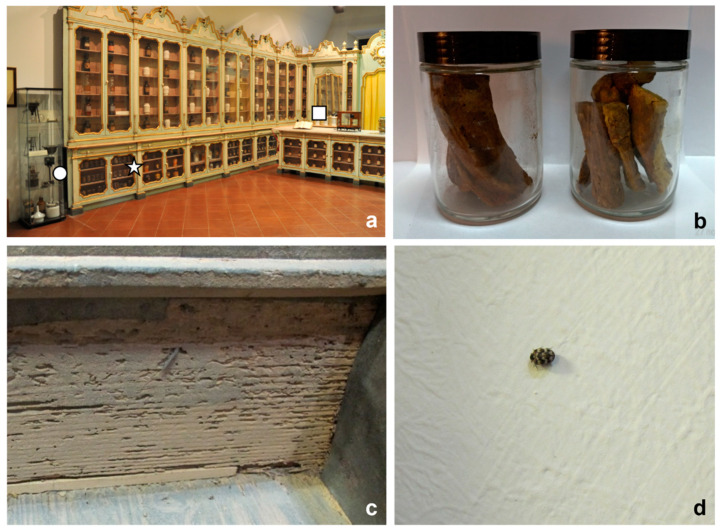
Analysis of insect biodeterioration in the Navarra-Bragliani Pharmacy: (**a**) points where insect samples were collected; (**b**) materials contained in two pharmacy jars found attacked by *Stegobium* *paniceum*. The position of the jars is indicated by a star in Figure 2a; (**c**) detail of the drawer indicated by a square in Figure 2a, showing damages by *Lyctus* *brunneus*; (**d**) live individual of *Anthrenus* *verbasci* walking in the position indicated by a circle in Figure 2a.

The surveys conducted on the equipment and furniture of the historical Navarra-Bragliani Pharmacy confirmed the presence and activity of biodeteriogenic insects. The presence of these insects was detected inside jars containing rhubarb and liquorice roots and inside an old drawer (Figure 2a,b). A live insect was found inside a cabinet on the north wall, near the jars. All these insect specimens were identified as belonging to *Stegobium* *paniceum* (Linnaeus) (Coleoptera: Anobiidae), together with associated traces such as body parts, wings, and wood dust.

*Stegobiumpaniceum*, commonly known as ‘drugstore beetle’ or ‘biscuit beetle’, is a cosmopolitan insect pest with polyphagous larvae that mostly feed on starch-based food (flour, bread, and cookies), spices, and inedible materials such as wool, paper, museum specimens. The species may also be found on pigeon nests [11,13,14,15,16]. Adults are 2.2–3.5 mm long [15] and reddish brown [12]. The female lays about 100 eggs [11] in the substrate, for an average of 3/4 generations per year [15]. Larvae are C-shaped, about 4 mm long, white with a yellow head [11].

Traces of floury wood dust and some elytra were also found inside an old damaged drawer, located under the marble top of the pharmacy counter (Figure 2a,c). The elytra were identified as belonging to *Lyctus* *brunneus* (Stephens) (Coleoptera: Bostrichidae). The powderpost beetle, *L. brunneus*, a cosmopolitan and synanthropic insect pest, mostly attacks hardwood in which the larvae dig tunnels. Adults are elongated and flat, about 7 mm long and dark brown. The female lays about 70 eggs on wooden pores and the life cycle variates from 2 to 18 months, according to temperature, humidity, and food source [17,18]. Larvae are C-shaped, covered by setae, and with a brown head [12].

Finally, one larva and some adults, all dead, were found on the floor of the pharmacy, near the cabinets on the north side. A live adult was also found on the north wall (Figure 2a,d). All these specimens were identified as belonging to *Anthrenus* *verbasci* (Linnaeus) (Coleoptera: Dermestidae). The varied carpet beetle, *A. verbasci*, a native of Eastern Europe, is now cosmopolitan [19]. Adults are round, 2–4 mm long, exhibiting black, white, brownish, and yellowish minute patches [11,12] and feeding on flower nectar and pollen. The female lays from 10 to 100 eggs and the life cycle variates from 6 to 12 months. The larvae are stout, 3–4 mm long [11], with the body covered by setae [11,12]. The larvae may be found in the wild in bird nests [20], but they may also damage items of animal origin, such as woollen carpets, textiles, taxidermy specimens (bird and mammal skin), insect collections, and animal glue [11,12,20]. The final location of the equipment and furniture of the Navarra-Bragliani Pharmacy is a room with carefully controlled temperature and humidity, and high museum standards of cleanliness, maintenance, and limited public access. Nevertheless, the presence of biodeteriogenic insects was detected, including a species that could seriously damage wood (*L. brunneus*) and another one that preferentially attacks books and organic materials contained in jars (*S. paniceum*). Another detected species (*A. verbasci*) may be attracted by insect residues, causing damages to parchment and other items of animal origin. The larvae of this species have also been reported causing allergic rhinitis [21].

Considering the destructivity of *L. brunneus*, although only fragments and not live species were found, a careful periodical monitoring of the furniture, wood pharmacy equipment, and of the entire room was programmed in order to prevent infestations.

As regards *S. paniceum*, the use of traps with stegobione was programmed, together with periodical surveys to prevent attacks by this species. For *A. verbasci*, all materials that could be a target of this species were carefully controlled, and periodical careful surveys were programmed for the furniture, pharmacy equipment, and the entire room, in order to prevent infestations.

The need to preserve and protect sites of historical and cultural relevance requires careful planning and monitoring to prevent and/or reduce damage from biodeteriogenic agents, according to the integrated pest management (IPM) strategy. The IPM involves biological, physical, and chemical methods for pest control that may reduce the use of environmentally unsafe pesticides. Developed for the first time around 1950 for the food industry, since 1980, it has been applied to museums and other cultural heritage sites [22].

In addition to the previously mentioned control of temperature and humidity in the exhibition room, associated with high cleanliness and maintenance standards, in the case of the Navarra-Bragliani Pharmacy, continuous monitoring of environmental conditions through the installation of a permanent ‘data logger’ would be useful, as would performing periodical surveys to check for presence and activity of biodeteriogenic insects. It is also relevant to seal hermetically the jars containing roots and plants, in order to prevent pest access and proliferation.

Studies on Italian historical pharmacies have been conducted thus far only from an artistic and cultural point of view, emphasising the various styles of equipment and furniture and the civic and social role of the pharmacy. Among those dating back to the 18th century and described in AISF ‘Calendari’ (including the Navarra-Bragliani Pharmacy), 24 are located in regions of northern Italy (7 in Emilia-Romagna, 7 in Piedmont, 5 in Veneto, 3 in Lombardy, 1 in Friuli-Venezia Giulia, and 1 in Liguria), 5 in central Italy (3 in Tuscany and 2 in Lazio), and 2 in southern Italy, in Campania [1]. The AISF is in the process of identifying and describing many more historical pharmacies, especially in southern Italy. However, no studies have been conducted to date on the state of conservation and the protection of ancient furniture and equipment of Italian historic pharmacies. The first study of this type on the Navarra-Bragliani Pharmacy in Ferrara may be useful for the monitoring and preservation of the precious 18th-century furnishing of this historical pharmacy, as well as for initiating similar studies on other ancient pharmacies, providing useful information to preserve these valuable cultural and scientific assets for the future.

## Data Availability

The data supporting this investigation are available from the cor-responding author [M.P.] upon reasonable request.
